# A two-component system MechNtrB/MechNtrC related to nitrogen metabolism regulation in *Micromonospora echinospora* DSM43816

**DOI:** 10.3389/fmicb.2025.1678324

**Published:** 2025-10-31

**Authors:** Yuxin Long, Jiayi Lu, Siyi Leng, Congsi Li, Haiyan Ni, Long Zou, Zhiming Wu, Zhong-er Long

**Affiliations:** Nanchang Key Laboratory of Microbial Resources Exploitation and Utilization from Poyang Lake Wetland, College of Life Sciences, Jiangxi Normal University, Nanchang, China

**Keywords:** *Micromonospora echinospora*, nitrogen metabolism, metabolic regulation, two-component system, MechNtrB/MechNtrC

## Abstract

The NtrB/NtrC two-component signal transduction system (TCS), predominantly found in Gram-negative bacteria, plays a crucial role in nitrogen metabolism regulation. Through BLASTP analysis of the complete proteome of *Micromonospora echinospora* DSM 43816 against the NtrB/NtrC database, homologous proteins were identified and designated as MechNtrB and MechNtrC. Subsequent *in vitro* expression and phosphorylation assays demonstrated that both proteins undergo phosphorylation, indicating a phosphoryl transfer mechanism for signal transduction between them. Yeast two-hybrid (Y2H) screening using selective media further verified specific *in vivo* protein–protein interactions between MechNtrB and MechNtrC. Quantitative real-time PCR (RT-qPCR) analyses revealed that overexpression of *MechNtrB* or *MechNtrC* gene significantly modulated the expression levels of key nitrogen metabolism genes, including those encoding glutamine synthetase (GS), glutamate synthase (GOGAT), and glutamate dehydrogenase (GDH). These collective findings establish that MechNtrB/MechNtrC constitutes a functional TCS in *M. echinospora* that directly regulates bacterial nitrogen metabolism.

## 1 Introduction

*Micromonospora echinospora* is a common species of actinobacteria belonging to the genus *Micromonospora*. As a Gram-positive, aerobic, spore-forming filamentous bacterium, its most notable characteristic is the production of a clinically important aminoglycoside antibiotic (Li et al., 2018; Xu et al., 2022; Ni et al., 2014; Meenavilli et al., 2008). In 1963, researchers isolated and purified bioactive components from the bacterial fermentation broth through extraction and column chromatography, identifying a group of structurally related aminoglycoside compounds (later named the gentamicin C complex). By 1970, gentamicin was introduced into clinical practice, becoming a key therapeutic agent for severe Gram-negative bacterial infections. This bacterium is a key organism in drug discovery and microbial biotechnology.

Nitrogen is essential for microbial growth and reproduction, serving as a fundamental component of proteins and other macromolecules. Microorganisms in diverse environments have evolved varied nitrogen assimilation strategies, with Glutamine Synthetase (GS) Glutamate Synthase (GOGAT) and Glutamate Dehydrogenase (GDH) pathways being the primary pathway for ammonia assimilation (Gunka and Commichau, 2012; Tiffert et al., 2011; Liu et al., 2022; Fisher and Wray, 2008).

The GS/GOGAT pathway exhibits a low affinity for ammonia but high energy efficiency, making it suitable for nitrogen-replete conditions. The reactions proceed as follows:


$${\mathrm{NH}}_{3}+\text{Glutamate}+\mathrm{ATP}\overset{\mathrm{GS}}{\to }\text{Glutamine}+\mathrm{ADP}+\mathrm{Pi}$$

$$\text{Glutamine}+\alpha -\text{ketoglutarate}+\text{NADPH}\overset{\text{GOGAT}}{\to }2\text{Glutamate}+{\text{NADP}}^{+}$$

In contrast, the GDH pathway displays high ammonia affinity but high energy cost, favoring operation under nitrogen-limiting conditions. The reaction is:


$${\mathrm{NH}}_{3}+\alpha -\text{ketoglutarate}+\mathrm{NAD}(P)H\overset{\mathrm{GDH}}{\to }\text{Glutamate}+\mathrm{NAD}{\left(P\right)}^{+}+H_{2}O$$

To adapt to environmental nitrogen fluctuations, microorganisms have developed three major regulatory mechanisms: the Ntr system for global regulation in Gram-negative bacteria, the GlnR transcription factor-dependent regulation in Gram-positive bacteria, and sRNA-mediated modulation of key enzyme activity (Prival et al., 1973; Prival et al., 1971; Liu et al., 2024; Livny et al., 2006; Romeo et al., 2012). These regulatory systems ensure microbial survival advantages under varying nitrogen conditions.

The two-component system (TCS) is a major signaling mechanism enabling bacteria and archaea to sense and respond to environmental changes. A typical TCS comprises a membrane-associated histidine kinase (HK) and a cytoplasmic response regulator (RR) (North et al., 2023; Nixon et al., 1986). Upon signal perception, the HK autophosphorylates on a conserved histidine residue, and then the phosphate group is transferred to a conserved aspartate residue on the RR, inducing its activation and subsequent regulation of downstream genes or cellular processes.

A classic example is the NtrB/NtrC system, a key regulator of bacterial nitrogen metabolism. Nitrogen, primarily available as ammonium, is assimilated into biomass via different pathways depending on its availability. Under nitrogen limitation, assimilation proceeds through the GS/GOGAT pathway, which is tightly regulated by the Ntr system. The central regulatory cascade is as follows: The signal of nitrogen starvation is perceived by UTase, leading to the uridylylation of the PII protein (forming PII-UMP). Uridylylated PII no longer interacts with NtrB, thereby unleashing NtrB’s autokinase activity. NtrB, encoded by *glnL*, autophosphorylates and then transfers the phosphate to NtrC (encoded by *glnG*). Phosphorylated NtrC (NtrC-P) functions as a transcriptional activator for σ^54^-dependent promoters, driving the expression of genes essential for nitrogen scavenging and assimilation. Concurrently, PII-UMP promotes the deadenylylation of GS by ATase, activating the GS enzyme to work in concert with the NtrC-P-induced genetic program.

Structurally, NtrB features an N-terminal sensor domain and a C-terminal kinase domain with conserved motifs for histidine phosphorylation and ATP binding (Shaza and Khattab, 2020; Atkinson and Ninfa, 1993; Stock et al., 1989). NtrC consists of an N-terminal receiver domain containing the phospho-accepting aspartate residue, and a C-terminal domain responsible for DNA binding and transcriptional activation (Kustu et al., 1989; Morett and Segovia, 1993; Drummond et al., 1986; Reitzer and Magasanik, 1983). Both proteins function as dimers to regulate cellular nitrogen metabolism (Ronson et al., 1987; Alvarez and Georgellis, 2023).

Therefore, this study was designed to systematically investigate the existence and function of an NtrB/NtrC-like TCS in *M. echinospora* DSM 43816, a known producer of gentamicin. Initially, the BLAST tool was employed to perform multiple sequence alignments between the whole-protein sequences of *M. echinospora* and a constructed NtrB/NtrC protein database, yielding the homologous proteins MechNtrB and MechNtrC. Subsequently, their phosphorylation function *in vivo* was confirmed through heterologous protein expression and phosphorylation assays, while their interaction was verified using yeast two-hybrid (Y2H) technology. Finally, by constructing *MechNtrB* and *MechNtrC* genes overexpression strains and combining quantitative real-time PCR (RT-qPCR) analysis, the expression differences of key nitrogen metabolism enzyme genes including GS, GOGAT, and GDH, were compared between the wild-type and overexpression strains. These findings functionally elucidate the role of this MechNtrB/MechNtrC TCS in regulating nitrogen metabolism in *M. echinospora*. Our findings aim to elucidate a previously unrecognized regulatory layer in *M. echinospora*, potentially bridging a conceptual gap in our understanding of nitrogen sensing in Gram-positive bacteria, and offering new insights into the link between primary metabolism and antibiotic production in this industrially relevant organism.

## 2 Materials and methods

### 2.1 Strains, plasmids, primers, and reagents

All bacterial strains used in this study are listed in Supplementary Table S1. The plasmids are documented in Supplementary Table S2, and all specific primers are listed in Supplementary Table S3. All chemical reagents were analytical grade, and all biological reagents were molecular biology grade. The media used in the study were prepared as follows:

LB medium for *E. coli* culture: 20 g of LB powder was dissolved in distilled water, the pH was adjusted to 7.4, the final volume was brought to 1 L with distilled water.

YEPD medium for yeast culture: 50 g of YEPD powder was dissolved in distilled water, the pH was adjusted to 6.5, the final volume was brought to 1 L with distilled water.

SD/-Trp: 0.67 g of YNB, 0.074 g of CSM-TRP, and 2 g of glucose were dissolved in distilled water (pH 5.8), the final volume was brought to 100 mL.

SD/-Trp-Leu: 0.67 g of YNB, 0.064 g of CSM-TRP-LEU, and 2 g of glucose were dissolved in distilled water, the pH was adjusted to 5.8, the final volume was brought to 100 mL.

SD/-Trp-Leu-His: 0.67 g of YNB, 0.062 g of CSM-TRP-LEU-HIS, and 2 g of glucose were dissolved in distilled water, the pH was adjusted to 5.8, the final volume was brought to 100 mL.

Gauze’s No.1 medium for *M. echinospora* culture: 22.5 g of powder was dissolved in deionized water, the final volume was brought to 1 L.

ISP2 medium for *M. echinospora* culture: 20 g of ISP2 powder was dissolved in deionized water, the final volume was brought to 1 L.

Once the medium is prepared, it should be immediately sterilized at 121 °C for 20 min, cooled to room temperature, and stored at 4 °C for later use.

### 2.2 Bioinformatics analysis

The NtrB and NtrC protein databases were retrieved and downloaded from the NCBI website. Using the BLAST tool, the whole-protein sequences of *M. echinospora* were aligned against the constructed protein databases for NtrB and NtrC, respectively. A cutoff E-value of 1e^−5^ was set for the output, ultimately identifying the homologous proteins MechNtrB and MechNtrC (where the prefix “M” denotes the genus *Micromonospora*, and “ech” represents the first three letters of the species epithet *echinospora*). The basic physicochemical properties of MechNtrB and MechNtrC proteins were analyzed using the Expasy ProtParam tool. Transmembrane domains and signal peptide distributions were predicted by HMMTOP v2.0 and SignalP 4.1. Homology modeling of tertiary structures was performed using SWISS-MODEL, with model quality assessed through Ramachandran plot analysis. Protein–protein docking and interaction analyses were conducted using ZDOCK Server and PDBePISA.

### 2.3 Heterologous expression and purification of MechNtrB and MechNtrC

Due to the high GC content of the genes encoding MechNtrB and MechNtrC (Supplementary Texts S1, S2), which may reduce heterologous expression levels, the gene sequences were codon-optimized based on the degeneracy of the genetic code (Supplementary Texts S3, S4). Since MechNtrB is a membrane protein, we truncated its transmembrane domain after confirming that phosphorylation was independent of this region. The truncated sequence is provided in the supplementary materials (Supplementary Text S3). The optimized sequences were then cloned into the pET-29a(+) vector containing a 6 × His tag using the restriction enzymes *Sal* I and *EcoR* V, generating the expression plasmids pET-29a(+)-*MechNtrB* and pET-29a(+)-*MechNtrC*.

The expression vectors, along with the empty vector (control), were transformed into *E. coli* BL21 (DE3). Positive transform ants were inoculated into 100 mL LB medium supplemented with 50 μg/mL kanamycin and cultured at 37 °C, 200 rpm until the OD_600_ reached 0.6–0.8. Protein expression was induced by adding isopropyl β-D-1-thiogalactopyranoside (IPTG) to a final concentration of 0.3 mM, and incubated at 16 °C, 140×*g* for 12 h. Following centrifugation at 5,000×*g*, the bacterial pellet was resuspended in 20 mM Tris–HCl buffer (pH 7.5) and subjected to ice-cooled ultrasonication for cell disruption. The lysate was then centrifuged at 12,000×*g* for 30 min at 4 °C to separate the supernatant and precipitate. Protein expression profiles were subsequently analyzed by SDS-PAGE.

For protein purification, the above supernatant was loaded onto a Ni-NTA affinity column to allow binding. A stepwise gradient elution was performed using pre-chilled Tris–HCl buffer containing 50–500 mM imidazole, and fractions were collected at each concentration. The optimal imidazole concentration for target protein elution was determined by SDS-PAGE. The corresponding fractions were pooled and dialyzed against 1 × PBS buffer at 4 °C for 48 h to remove salts. The purified protein was stored at −20 °C for further use.

### 2.4 Phosphorylation detection of MechNtrB and MechNtrC

The purified MechNtrB and MechNtrC proteins, along with molecular weight markers, were separately loaded onto two identical SDS-PAGE gels. Following electrophoresis, one gel was subjected to Coomassie Brilliant Blue (CBB) staining to verify protein molecular weights and band positions; the parallel gel was processed with methyl green staining for phosphorylation detection.

### 2.5 Yeast two-hybrid analysis

The DNA fragments of *MechNtrB* and *MechNtrC* were amplified by PCR using sequence-specific primers, with plasmids pET-29a(+)-*MechNtrB* and pET-29a(+)-*MechNtrC* serving as templates, respectively. After fragment purification, the amplified *MechNtrB* fragment was cloned into the pGBT9 vector using *Sal* I/*Not* I restriction sites to generate the recombinant plasmid pGBT9-*MechNtrB*. Similarly, the *MechNtrC* fragment was inserted into the pGAD10 vector using *Xho* I/*EcoR* I sites to construct pGAD10-*MechNtrC*.

In this part, all yeast competent cell preparations were performed using the lithium acetate/single-stranded carrier DNA/polyethylene glycol (LiAc/SS-DNA/PEG) method. The plasmids pGBT9, pGBT9-*MechNtrB*, and pGBT9-*AK2* were independently transformed into AH109 competent yeast cells via the lithium acetate (LiAc)-mediated heat shock method. The positive transformants, labeled as AH109/pGBT9, AH109/pGBT9-*MechNtrB*, and AH109/pGBT9-*AK2* respectively, were selected on SD/-Trp plates incubated at 30 °C for 2–3 days. Subsequently, the pGAD10 plasmid was introduced into AH109/pGBT9 cells, pGAD10-MechNtrC into AH109/pGBT9-*MechNtrB* cells, and pGAD10-*AIF* into AH109/pGBT9-*AK2* cells, with selection performed on SD/-Leu-Trp plates. Finally, the transformants AH109/(pGBT9/pGAD10), AH109/(pGBT9-*MechNtrB*/pGAD10-*MechNtrC*), and AH109/(pGBT9-*AK2*/pGAD10-*AIF*) were culture preservation after cultivation, then streaked onto SD/-His-Leu-Trp plates to assess growth phenotypes. Here, proteins AK2/AIF, known to have strong interactions, were used as a positive control.

### 2.6 Construction of MechNtrB/MechNtrC overexpression strains

To determine the antibiotic sensitivity of *M. echinospora*, glycerol stocks were streaked onto agar plates containing gradient concentrations (12.5, 25, 37.5, and 50 μg/mL) of selected antibiotics: apramycin, kanamycin, chloramphenicol, ampicillin, and nalidixic acid. The plates were incubated inverted at 30 °C for 5–7 days, with daily observation of bacterial growth characteristics.

Gene fragments encoding native *MechNtrB* and *MechNtrC* were amplified by PCR using *M. echinospora* genomic DNA as template with specifically designed primers. The amplified products were cloned into the pSET152AKEx vector using *Nde* I/*Xba* I restriction sites to generate recombinant plasmids pSET152AKEx-*MechNtrB* and pSET152AKEx-*MechNtrC*. These constructs were subsequently transformed into *E. coli* ET12567 (pUZ8002) as donor strains. For intergeneric conjugation, recipient *M. echinospora* cells were cultured to mid-log phase. Donor and recipient cells were mixed at a 1:1 ratio and allowed to mate for 40 min before plating onto Gauze’s No. 1 medium supplemented with Mg^2+^. After 22 h of incubation at 30 °C, plates were overlaid with sterile water containing 50 μg/mL apramycin and 25 μg/mL nalidixic acid. This procedure yielded the *MechNtrB*- and *MechNtrC*-overexpressing strains, respectively.

### 2.7 RT-qPCR

Gene-specific primers for *M. echinospora* were designed using its genomic DNA as a template to quantify the expression of key nitrogen metabolism genes, including the GS, GOGAT, and GDH genes. The 16S rRNA gene was used as an endogenous reference for normalization.

The wild-type strain of *M. echinospora* and its derivative strains overexpressing MechNtrB/MechNtrC were cultured in 50 mL of ISP2 liquid medium (3 biological replicates) at 30 °C with constant shaking for 72 h. Bacterial cells were harvested by centrifugation at 8,000×*g* for 10 min at 4 °C. The cell pellets were transferred to pre-cooled mortars and rapidly ground under liquid nitrogen. The resulting powder was immediately transferred to 1.5 mL RNase-free micro centrifuge tubes, and 1 mL of RNAiso™ Plus reagent (Takara) was added for cell lysis. The samples were vortexed vigorously for 30 s to ensure complete homogenization, followed by incubation on ice for 5–10 min to facilitate nucleic acid release. After adding 200 μL of chloroform (20% of the lysate volume), the mixtures were shaken vigorously for 15 s to achieve complete emulsification. Following 3 min of incubation at room temperature, phase separation was performed by centrifugation at 11,000×*g* for 20 min at 4 °C. The upper aqueous phase containing RNA (approximately 50% of total volume) was carefully collected. First-strand cDNA was synthesized from the extracted RNA using the PrimeScript RT Master Mix (Takara) according to the manufacturer’s protocol, and the resulting cDNA was stored at −20 °C.

RT-qPCR was performed using TB Green™ Premix Ex Taq™ II (Tli RNaseH Plus) (Takara) following the manufacturer’s instructions. The reaction mixture composition is shown in Table 1, and the reaction procedure for qRT-PCR is shown in Table 2. Relative quantification of target gene expression was calculated using the comparative ΔΔCt method. Analysis of significant differences was performed with SPSS software (Version IBM SPSS Statistics 27), tested by one-way analysis of variance (ANOVA). *p* < 0.05 was considered to indicate a statistically significant difference.

**Table 1:** RT-qPCR reaction system.

Reagent	Reagent
TB Green Premix Ex Taq II (Tli RNaseH Plus) (2×)	10.0
ROX Reference Dye II (50×)	0.4
PCR Forward Primer (10 μM)	0.8
PCR Reverse Primer (10 μM)	0.8
Template cDNA	2.0
ddH_2_O	6.0
Total	20

**Table 2:** RT-qPCR reaction procedure.

Operations	Temperature (°C)	Time (sec)	Cycle
Pre-denaturation	95	30	
Amplification	95	5	40
	60	34	
	95	15	
Melting	60	60	
	95	15	

## 3 Results

### 3.1 Structural analysis and molecular docking of MechNtrB/MechNtrC

After comparison and screening, MechNtrB/MechNtrC was found to be the most homologous protein in *M. echinospora* to the NtrB/NtrC systems, respectively.

Bioinformatic analysis revealed that MechNtrB is a 482-amino acid hydrophobic protein containing two transmembrane domains but lacking signal peptide sequences. MechNtrC comprises 248 hydrophobic amino acids with neither transmembrane regions nor signal peptides (Supplementary Figure S1).

The MechNtrB protein was modeled using the histidine kinase of *Micromonospora haikouensis* as a template (coverage = 1.0, GMQE = 0.84) (Supplementary Figure S2), while MechNtrC was modeled based on the response regulator of *Mycobacterium tuberculosis* (coverage = 0.93, GMQE = 0.78) (Supplementary Figure S2). Ramachandran plot analysis revealed that 95.1% of MechNtrB and 92.0% of MechNtrC residues were in favored regions, with only 0.0 and 0.1% in disallowed regions, respectively (Supplementary Figure S2). These results confirm the reliability of the models for subsequent molecular docking studies.

**Figure 1:**
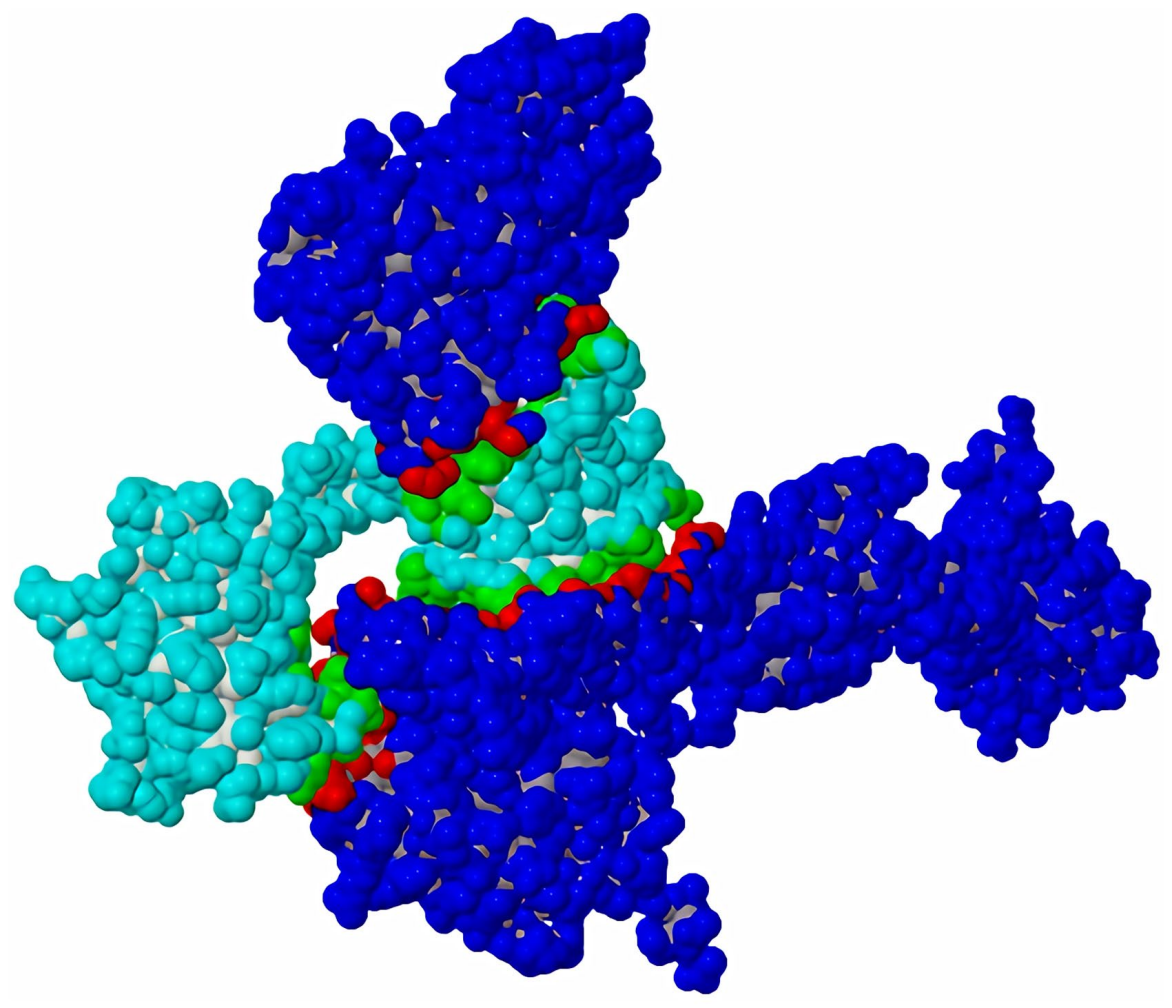
The molecular docking results between MechNtrB and MechNtrC proteins.

Molecular docking analysis demonstrated a specific interaction mode between the two proteins, featuring an extensive interface (13,544 Å^2^) with strong binding affinity (ΔG = −11.5 kcal/mol, *p*-value < 0.5). The interaction was stabilized by 16 hydrogen bonds (each contributing ~0.5 kcal/mol) and 13 salt bridges (each contributing ~0.3 kcal/mol), indicating a specific binding pattern (Figure 1).

### 3.2 MechNtrB and MechNtrC protein expression, purification and phosphorylation detection

**Figure 2:**
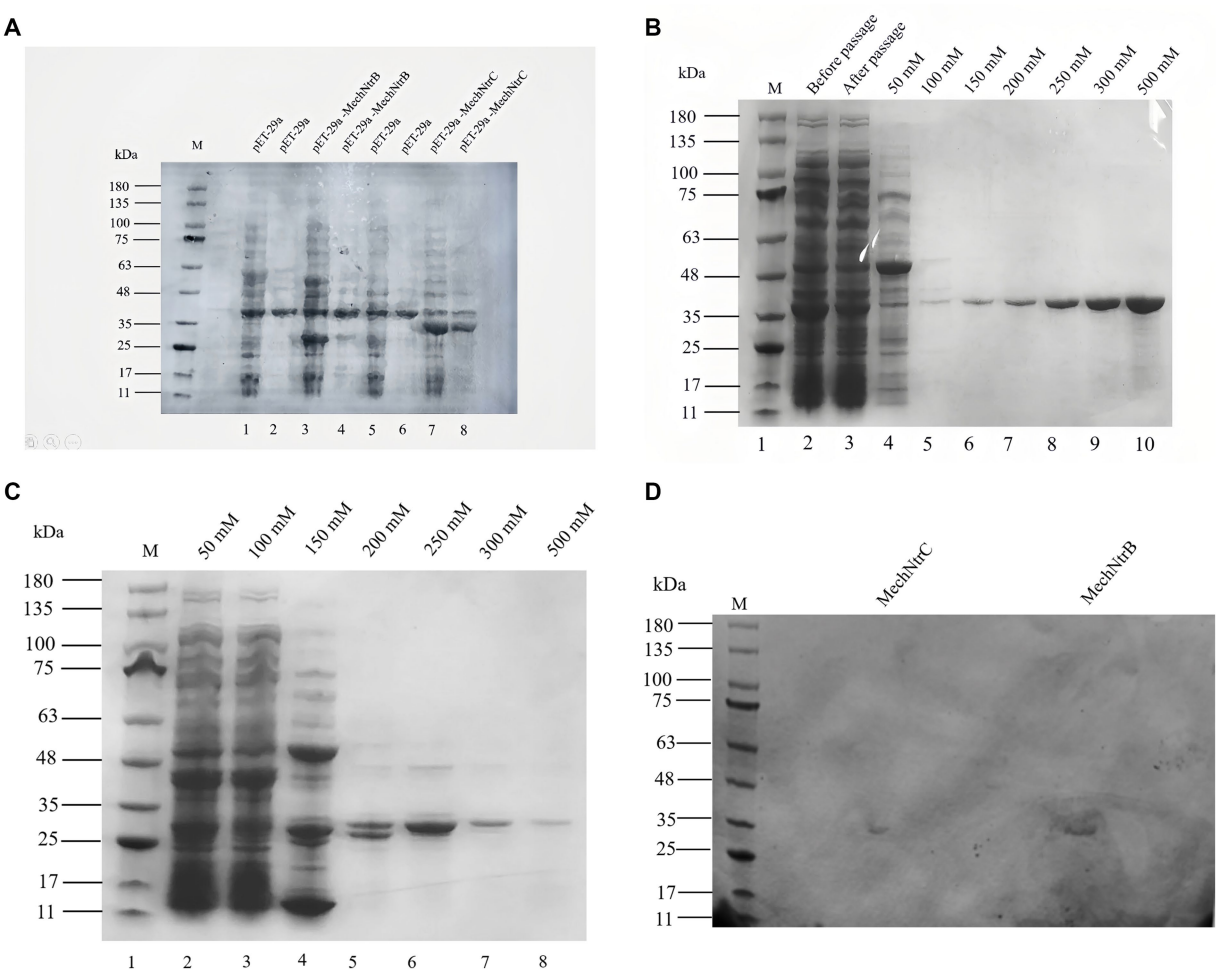
Expression, purification, and phosphorylation detection of MechNtrB and MechNtrC. **(A)** SDS-PAGE analysis of protein expression after induction and sonication. Lanes 1, 3, 5, and 7: soluble supernatant; Lanes 2, 4, 6, and 8: insoluble precipitate. **(B)** Purification of MechNtrB. Lanes 4–10: elution fractions with different imidazole concentrations. **(C)** Purification of MechNtrC. Lanes 1–8: elution fractions with varying imidazole concentrations. **(D)** Phosphorylation detection by methyl green staining after electrophoresis.

Expression and purification of MechNtrB and MechNtrC were systematically analyzed. SDS-PAGE detection showed that clear bands of expected molecular weight were observed in the protein supernatant of the experimental group, which confirmed that the two proteins were successfully expressed in the expression system of *E. coli* BL21 (Figure 2A). Subsequently, SDS-PAGE analysis of the eluted fractions with different imidazole concentrations showed that MechNtrB protein achieved the best elution effect at 500 mM imidazole concentration (Figure 2B), and MechNtrC protein showed the highest elution efficiency at 250 mM imidazole concentration (Figure 2C).

Two pieces of protein gel were, respectively, stained with Coomassie brilliant blue (Supplementary Figure S4) and methyl green (Figure 2D). Phosphorylation detection using methyl green staining revealed distinct green bands for both MechNtrB and MechNtrC at their expected molecular weights, demonstrating that these proteins undergo phosphorylation in *E. coli* BL21.

### 3.3 Yeast two-hybrid analysis

**Figure 3:**
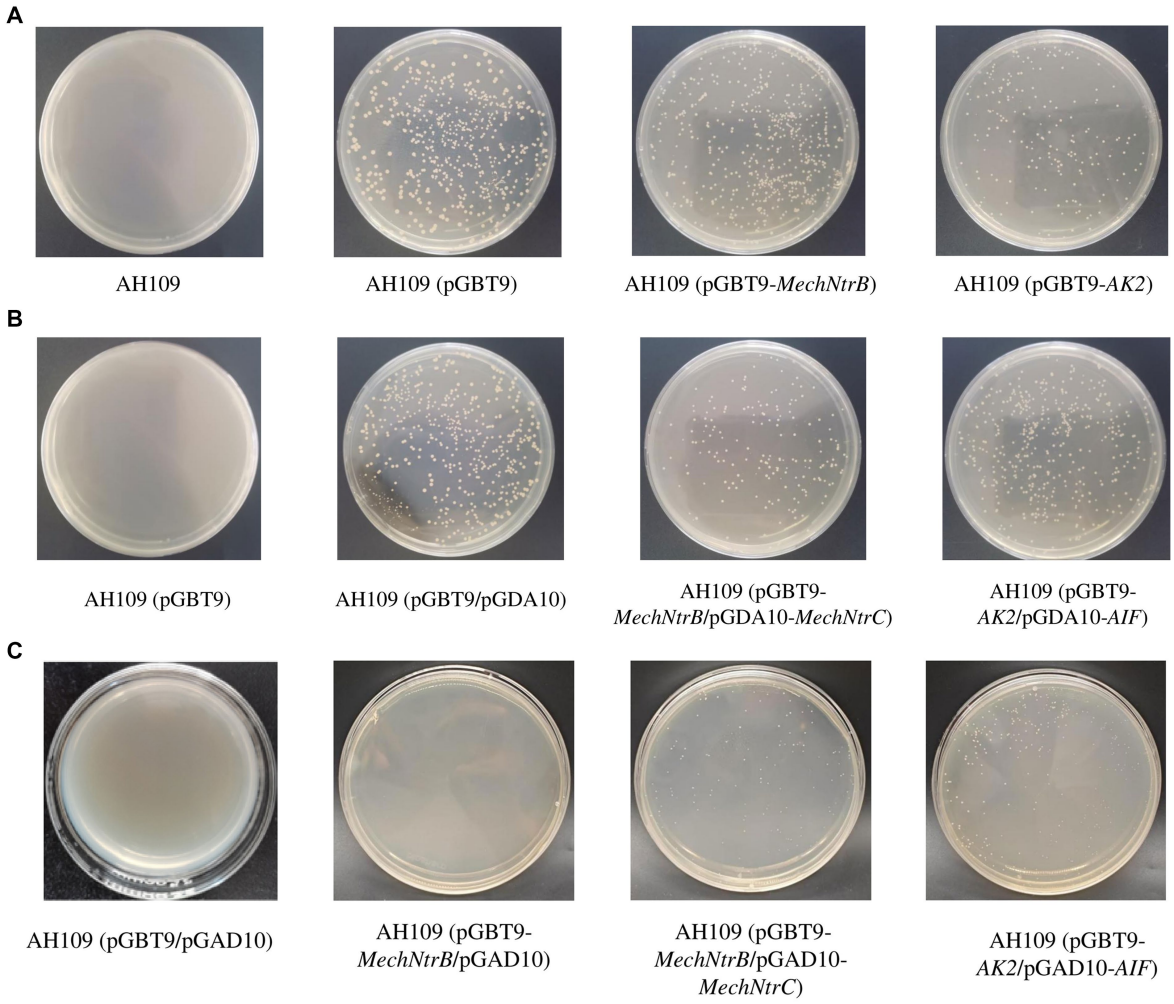
Growth state of yeast AH109 on different defect media. **(A)** SD/-Trp medium. **(B)** SD/-Trp/-Leu medium. **(C)** SD/-Trp/-Leu/-His medium.

Yeast two-hybrid screening demonstrated successful protein–protein interaction between MechNtrB and MechNtrC. Initial selection on SD/-Trp medium confirmed transformation efficiency (Figure 3A), with all bait vector-containing strains (AH109/pGBT9, AH109/pGBT9-*MechNtrB*, and AH109/pGBT9-*AK2*) growing while untransformed AH109 failed. Subsequent dual selection on SD/-Leu-Trp medium verified co-transformation of bait and prey vectors (Figure 3B), with growth observed for all double transform ants, including AH109/(pGBT9/pGAD10), AH109/(pGBT9-*MechNtrB*/pGAD10-*MechNtrC*), and AH109/(pGBT9-*AK2*/pGAD10-*AIF*). Critical interaction testing on SD/-His-Leu-Trp medium showed specific growth of both AH109/(pGBT9-*MechNtrB*/pGAD10-*MechNtrC*) and (AH109/pGBT9-*AK2*/pGAD10-*AIF*), while the negative control AH109/(pGBT9/pGAD10), and others, such as AH109/(pGBT9-*MechNtrB*/pGAD10) and AH109/(pGBT9/pGAD10-*MechNtrC*), failed to grow, confirming the specific interaction between MechNtrB and MechNtrC through reporter gene *his* activation (Figure 3C).

### 3.4 RT-qPCR

After various antibiotic susceptibility tests at different concentrations, the results demonstrated that *M. echinospora* could grow on plates containing kanamycin and nalidixic acid, but failed to grow on plates supplemented with apramycin. The specific results are shown in Table 3.

**Table 3:** Antibiotic sensitivity test of *M. echinospora* DSM43816.

Antibiotic	0 μg/mL	12.5 μg/mL	25 μg/mL	37.5 μg/mL	50 μg/mL
Kanamycin	++	++	++	+	+
Chloramphenicol	++	−	−	−	−
Apramycin	++	−	−	−	−
Ampicillin	++	−	−	−	−
Nalidixic acid	++	++	++	+	+

**Figure 4:**
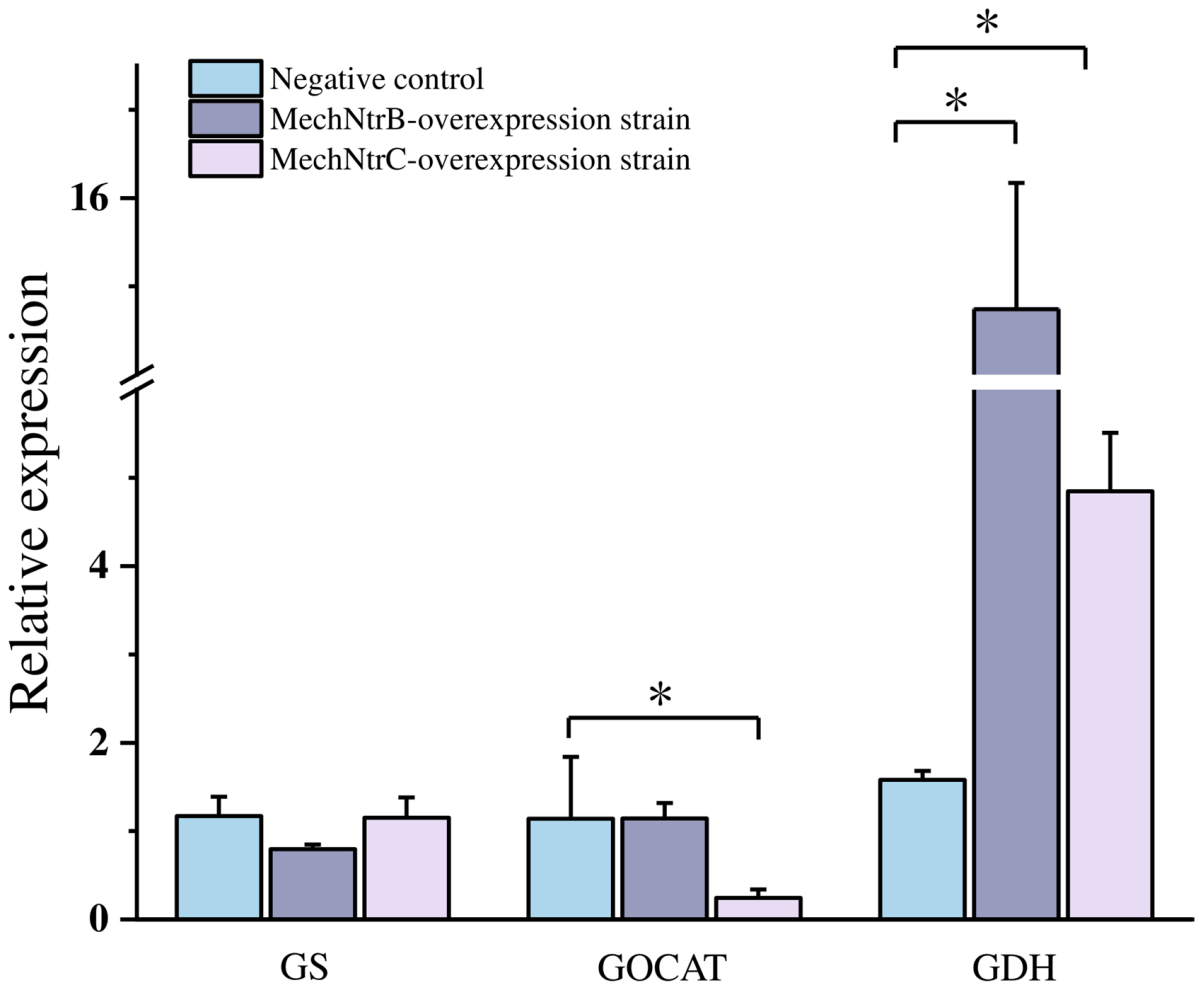
Expression levels of key enzyme genes in nitrogen metabolism, a *p* value < 0.05 indicates a significant difference.

The impact of MechNtrB or MechNtrC overexpression on the transcription of key nitrogen metabolism genes was assessed by qRT-PCR (Figure 4), overexpression of either gene resulted in a significant increase in glutamine synthetase gene, whereas it decreased the expression of the glutamate synthase and glutamate dehydrogenase genes.

## 4 Discussion

The TCS is a core regulatory mechanism for bacteria to sense and respond to environmental changes (Cao et al., 2024). Research on its functions is of great significance for understanding bacterial environmental adaptation mechanisms, revealing pathogenic mechanisms of pathogenic bacteria, and developing novel antibiotic targets (Ronson et al., 1987; Guo et al., 2024). Among these systems, NtrB/NtrC, as the first discovered TCS in bacteria, has been confirmed to participate in nitrogen metabolism regulation and is primarily distributed in Gram-negative bacteria such as *E. coli*, *Klebsiella pneumoniae*, and *Salmonella typhimurium*. However, studies on whether functional Ntr systems exist in Gram-positive bacteria, particularly within the genus *Micromonospora* which is renowned for its significant biosynthetic potential, remains largely unexplored (Schaefers et al., 2021; Shaza and Khattab, 2020; Nixon et al., 1986). Prior to this study, the McarNtrB/McarNtrC system in *M. carbonacea* JXNU-1 reported by our laboratory (Xiang et al., 2025) represented a rare exception, with no other homologous systems documented in this genus.

**Figure 5:**
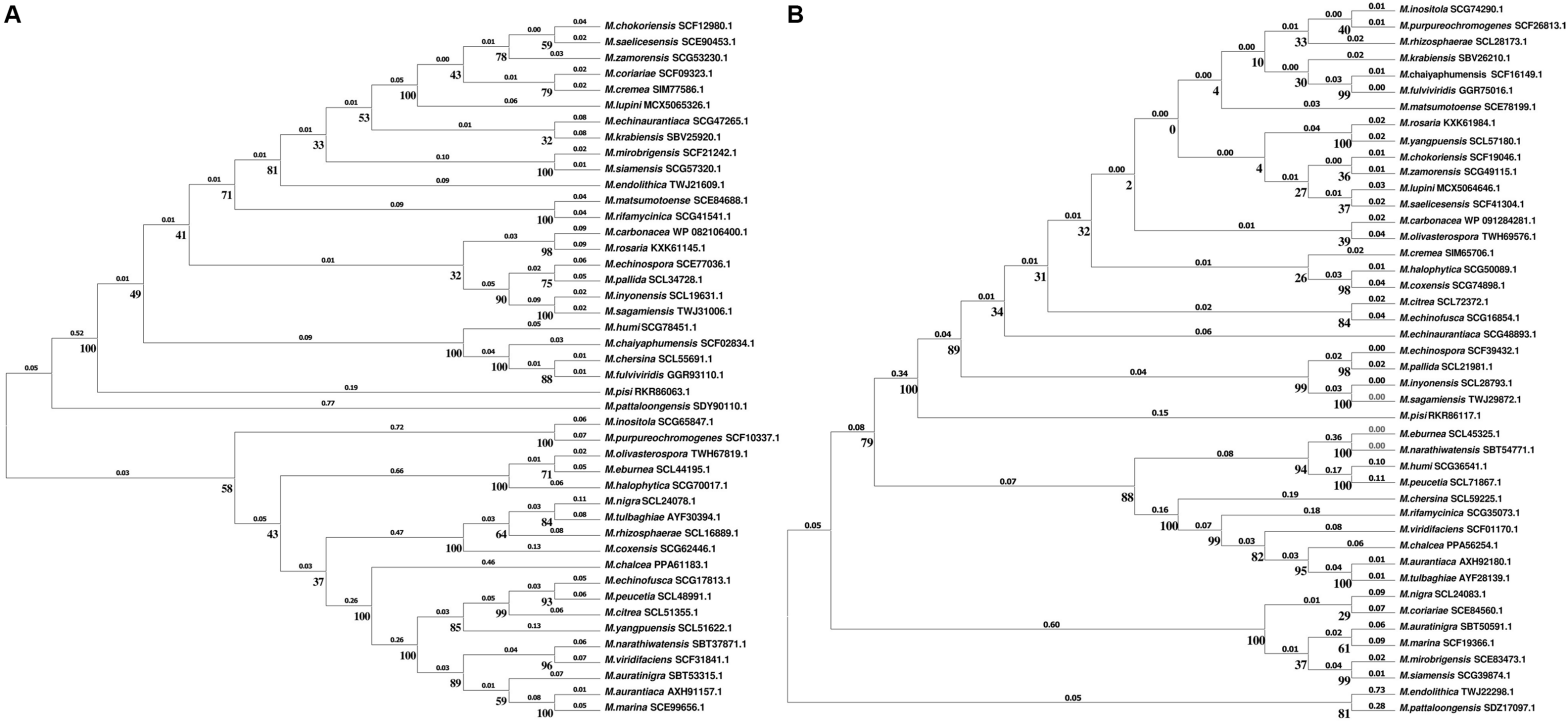
**(A)** Phylogenetic tree of *Micromonospora* based on MxxxNtrB. **(B)** Phylogenetic tree of *Micromonospora* based on MxxxNtrC.

Building upon this background, we identified NtrB/NtrC homologous proteins, MechNtrB/MechNtrC, in *M. echinospora* through bioinformatics analysis. This discovery prompted a broader investigation into the distribution of the Ntr system across the genus *Micromonospora*, especially given the genus’s capacity to synthesize structurally diverse secondary metabolites with antibacterial, antitumor, and antiviral activities, highlighting its considerable potential for pharmaceutical and agricultural applications (Silva de et al., 2024; Hifnawy et al., 2020; Qiu et al., 2008; Mazmancı et al., 2023). Our genomic survey revealed that homologous proteins MxxxNtrB/MxxxNtrC were present in all 44 *Micromonospora* strains with available genome sequences (where the prefix “M” represents the genus *Micromonospora* and “xxx” denotes the first three letters of the species epithet), suggesting this system is widely conserved within the genus, as further illustrated by the phylogenetic analysis (Figure 5). We first retrieved protein sequences of “nitrogen regulator II NtrB” and “nitrogen metabolism transcriptional regulator NtrC” from Gram-negative bacteria in the NCBI protein database. Concurrently, complete genome (protein) sequences of all *Micromonospora* actinomycetes were downloaded from the NCBI whole-genome sequence database. Local BLASTP alignments were then performed between the retrieved NtrB/NtrC components and the complete protein sequences of *Micromonospora* actinomycetes, with an E-value cutoff set at 1e^−5^. Notably, homologous proteins MxxxNtrB/MxxxNtrC were identified in all 44 *Micromonospora* actinomycetes with publicly available genome (protein) sequences. Upon examining the phylogenetic trees of MxxxNtrB and MxxxNtrC, we observed distinct topological structures between the two, suggesting that they may have been subjected to different selective pressures during evolution. This divergence implies that in certain lineages, MxxxNtrC might engage in functional interactions with regulatory systems beyond its canonical partnership with MxxxNtrB.

Having established the widespread presence of Ntr homologs, we next sought to characterize the basic characteristics of the MechNtrB/MechNtrC pair. The defining characteristics of a typical TCS include specific protein–protein interaction and the capacity for autophosphorylation and phosphate transfer. Our bioinformatic analysis of MechNtrB and MechNtrC from *M. echinospora* predicted these properties, which were subsequently confirmed experimentally.

Specifically, phosphorylation assays demonstrated that both MechNtrB and MechNtrC undergo phosphorylation in *E. coli* BL21. In Gram-negative bacteria, the NtrB/NtrC system has been extensively studied for its role in nitrogen metabolism regulation through phosphorylation cascades. For instance (Keener and Kustu, 1988), demonstrated that in *E. coli*, NtrB undergoes autophosphorylation, whereas NtrC can only be phosphorylated or dephosphorylated in the presence of NtrB. Similarly (Cullen et al., 1996), identified RcNtrB and RcNtrC in the photosynthetic bacterium *Rhodobacter capsulatus* as putative members of a two-component system. Their study revealed that RcNtrB undergoes autophosphorylation *in vitro*, while RcNtrC is specifically phosphorylated by RcNtrB *in vivo*. Notably, RcNtrC appears to be a dedicated phosphatase for RcNtrB, as it is not phosphorylated by low-molecular-weight phosphodonors or *Salmonella typhimurium* NtrB (StNtrB). In this study, heterologous expression and in vitro phosphorylation assays of *M. echinospora* MechNtrB/MechNtrC in *E. coli* BL21 revealed that both proteins could undergo phosphorylation. This observation, however, must be interpreted with consideration of a potential confounding factor: the presence of the endogenous NtrB/NtrC system in the *E. coli* BL21 expression host. It is possible that MechNtrC phosphorylation was mediated, at least in part, by the host’s NtrB, suggesting that the phosphotransferase activity might not be strictly species-specific. Nevertheless, the functional conservation of MechNtrB/MechNtrC, despite *Micromonospora* being Gram-positive, implies an evolutionary retention of this regulatory mechanism.

Beyond phosphorylation, protein–protein interaction is another hallmark of TCSs. Molecular docking predicted, and Y2H assays confirmed, a robust and specific interaction between MechNtrB and MechNtrC. This finding aligns with previous studies in Gram-negative bacteria, such as the interaction between NtrB and NtrC in *Klebsiella pneumoniae* demonstrated by Martínez-Argudo et al. (2001), and strongly supports the signal transduction functionality of the MechNtrB/MechNtrC system in *M. echinospora*. Collectively, the phosphorylation and interaction data confirm that MechNtrB/MechNtrC exhibits the hallmark features of a canonical TCS.

The function of NtrB/NtrC in Gram-negative bacteria has been widely studied. As early as Reitzer, (2003) elucidated the classical framework of complex nitrogen metabolism regulation mediated by NtrB/NtrC in *E. coli*. Under nitrogen-sufficient conditions, the GDH pathway dominates, and uridylyl-removing enzyme (UR) removes UMP from PII/GlnK, thereby activating the phosphatase activity of NRII (NtrB) and suppressing the expression of Ntr-regulated genes. Conversely, under nitrogen-limiting conditions, uridylyltransferase (GlnUTase) promotes the uridylylation of PII/GlnK, which inhibits the phosphatase activity of NtrB, leading to the accumulation of phosphorylated NtrC (NtrC~P), and then activates σ^54^-dependent transcription of nitrogen-regulated genes. This classical regulatory framework of nitrogen metabolism is also applicable to various other Gram-negative bacteria. Subsequent studies by various researchers have revealed that the NtrB/NtrC system not only regulates nitrogen metabolism but is also frequently associated with bacterial biofilm formation, invasiveness, and virulence. For instance, Alford et al. (2020) constructed *Pseudomonas aeruginosa* mutants (Δ*ntrB*, Δ*ntrC*, and Δ*ntrBC*) and observed that the Δ*ntrBC* double mutant exhibited a > 40% reduction in biofilm formation. RNA-Seq analysis of the deletion mutants defined their transcriptional profiles, uncovering a significant correlation between NtrB/NtrC and virulence factors. Similarly Kukolj et al. (2020), conducted proteomic and metabolomic analyses of *Azospirillum brasilense ntrC* mutants under high- and low-nitrogen conditions. Their findings demonstrated that NtrC not only plays a pivotal role in nitrogen metabolism but also participates in the synthesis of proteins involved in antioxidant stress responses (e.g., glutathione S-transferase and hydroperoxide resistance proteins). This highlights the importance of NtrC in bacterial survival under oxidative stress conditions. In this study, to directly assess the regulatory role of MechNtrB/MechNtrC in nitrogen metabolism, we constructed overexpression strains and analyzed the expression of key nitrogen assimilation genes (GS, GOGAT, and GDH). The significant alterations in gene expression upon overexpression of either MechNtrB or MechNtrC firmly establish their involvement in regulating nitrogen metabolism in *M. echinospora*. This finding in a Gram-positive actinomycete is particularly intriguing because nitrogen metabolism in these bacteria is typically governed by the GlnR regulator. The coexistence of a NtrB/NtrC homologous system suggests the potential for a more complex, dual-track regulatory mechanism (e.g., synergy or complementarity between GlnR and NtrB/NtrC), which raises compelling questions for future research. For instance, is the regulatory network in *M. echinospora* analogous to the classical NtrBC framework in *E. coli*? Furthermore, given that *M. echinospora* is a critical producer of gentamicin, an important question arises: could the MechNtrB/MechNtrC system influence antibiotic yield by modulating nitrogen metabolism, which is often linked to secondary metabolite biosynthesis? Further experiments are required to validate this possibility.

Our findings present a more comprehensive and systematic analysis than the McarNtrB/McarNtrC system studies in *M. carbonacea* by investigating the existence and evolutionary relationships of the ntrB/ntrC system from a genus-wide perspective in *Micromonospora*, leading to more profound conclusions. The above results can provide a basis for revealing the molecular mechanism research of nitrogen metabolism regulation in *M. echinospora*, and also lay the foundation for subsequent exploration of the influence of nitrogen sources on the synthesis of secondary metabolites in *M. echinospora*.

## 5 Conclusion

The NtrB/NtrC TCS is a pivotal regulator of nitrogen metabolism, historically characterized in Gram-negative bacteria. While a homologous system was recently identified in *M. carbonacea*, its prevalence and functionality across the genus remained largely unexplored. This study provides compelling evidence that expands upon this initial finding. We first confirmed that NtrB/NtrC homologs are not an isolated occurrence but are widespread across the *Micromonospora* genus. Through a comprehensive characterization of the MechNtrB/MechNtrC system in *M. echinospora*—integrating bioinformatics, molecular docking, heterologous expression, phosphorylation assays, yeast two-hybrid screening, and gene overexpression—we definitively established its function in directly modulating key nitrogen metabolic genes. This work significantly strengthens the evidence for a functional NtrB/NtrC-like TCS in Gram-positive bacteria. Future research should employ genetic knockouts and metabolomics to decipher the precise regulatory network and explore its potential in modulating the synthesis of valuable secondary metabolites, such as gentamicin.

## Data Availability

The original contributions presented in the study are included in the article/Supplementary material, further inquiries can be directed to the corresponding author.
