# Supplementary material for: A two-component system MechNtrB/MechNtrC related to nitrogen metabolism regulation in Micromonospora echinospora DSM43816
Source: Front Microbiol. 2025 Oct 31;16:1678324. doi: 10.3389/fmicb.2025.1678324 (PMC12616635; doi:10.3389/fmicb.2025.1678324)
Supplement: Supplementary file 1 [file Table_1.DOCX]

**Supplementary Materials**

# **A two-component MechNtrB/MechNtrC system related to nitrogen metabolism regulation in *Micromonospora echinospora* DSM43816**

Yuxin Long, Jiayi Lu, Siyi Leng, Congsi Li, Haiyan Ni, Long Zou, Zhiming Wu, Zhong-er Long*

Nanchang Key Laboratory of Microbial Resources Exploitation & Utilization from Poyang Lake Wetland, College of Life Sciences, Jiangxi Normal University, Nanchang, China

**Correspondence**: Zhong-er Long, Nanchang Key Laboratory of Microbial Resources Exploitation & Utilization from Poyang Lake Wetland, College of Life Sciences, Jiangxi Normal University, Nanchang, China. E-mail: [longzhonger@163.com](mailto:longzhonger@163.com)

## **Text S1** Original sequence of MechNtrB after truncating the transmembrane region

ACCCGGGTCACCGAGCTGCCACTGGACCGGGGCGAGGTCGCCCTCTCCGTCCGGGTGCCGGCCGCCGACACCGACCCGCGCACCGAGGTCGGCCAGGTCGGCGTCGCGCTCAACCGGATGCTCGGGCACGTCGCCGCCGCGCTCGCCGCCCGCCAGGCCAGCGAGACCCGGGTACGCCAGTTCGTCGCCGACGCCAGCCACGAGCTGCGTACCCCGCTCGCCGCGATCCGGGGGTACGCGGAGGTGGCCCGCCGGGGCCGCGCGCAGGCCCCGCCCGACGTGGCGCACGCGCTGCGCCGGGTGGAGTCGGAGAGCGTGCGGATGACCAGCCTCGTGGACGACCTGCTGCTGCTGGCCCGACTCGACTCGGGACGACCGCTGGTGTCCGAGCCGGTCGACCTGTCCGCGCTGGTGGTCGACGCGGTCAGCGACGCGCACGTCGCCGGTCCCGAGCAGCGCTGGCAGCTCGACCTGCCGGACGAGGCGGTCGAGGTGTCGGGCGACGCCGCCCGGCTGCACCAGGTGGTGACCAACCTGCTGACCAACGCGCGGGTGCACACTCCGCCCGGCACCACCGTCACCACCACGCTGCGGGCCGAGCCGGACACCGCCGTGCTGACCGTCGCCGACGACGGGCCCGGTATCCCGGCGGAACTGCAACCGGAGGTGTTCGAACGGTTCGCCCGGGGCGACAGCTCCCGCTCCCGGGCGCACGGCAGCACCGGCCTTGGACTGGCCATCGTGGCCGCCGTGGTGGACGCCCACCACGGTCGGGTCGAGGTGGACAGCCGACCCGGGCGGACCCTGTTCACCGTACGTCTGCCGGTTCACACCGGTGCTACTACCTC

TGCA

## **Text S2** Original sequence of MechNtrC

ATGGTGACCGACGGGCATGCGTTGCCGGGCCGGGTCGAGCTGCGCCGCCCGGACGGCGGGCCGGTCCGCGTGCTGGTGGTCGACGACGAGCCCACCCTGACCGACCTGCTCGCCATGGCGCTGCGCTACGAGGGCTGGCAGGTGGCCACCGCCGGCAACGGCATGGCCGCGCTGAGCAGCGCCCGGCAGGTCCGCCCGGACGTGGTGGTGCTCGACGTCATGTTGCCCGACCTGGACGGCTTCCAGGTGCTCCGCCGGCTGCGCGAGCAGTCGCCGAACGTGCCGGTGCTCTTCCTGACCGCCCGGGACGCGGTGCAGGACCGGATCGCCGGGCTGACCGTCGGCGGCGACGACTACGTCACCAAGCCGTTCAGCCTGGAGGAGGTGATCGCCCGGCTCCGGGCGCTGCTGCGCCGTGCCGGGCTGGCCGTGGCCGCCCGCGAGGACGCCGTCCTCACCGTCGGCGACCTGAGCCTCGACGAGGACAGCCACGAGGTCCGCCGGGGGGACGACCTGATCACCCTCACCGCCACCGAGTTCGAGCTGCTCCGCTACCTGATGCGCAACCCCCGCCGGGTGCTCAGCAAGGCCCAGATCCTCGACCACGTCTGGAACTACGACTTCGGCGGGCAGGCGAACGTCGTCGAGCTGTACATCTCGTACCTCCGGAAGAAGATCGACGCCGGCCGCGAACCGATGATCC

ACACGTTGCGTGGGGCGGGGTATGTCCTCAAGCCGGCCCCG

## **Text S3** Optimized sequence of MechNtrB after truncating the transmembrane region

ACTCGTGTAACCGAATTGCCGCTGGACCGTGGTGAAGTTGCGCTGTCCGTTCGTGTTCCAGCGGCTGATACCGATCCGCGTACTGAAGTTGGTCAGGTTGGTGTTGCACTGAACCGTATGCTGGGTCACGTAGCAGCTGCACTGGCTGCACGTCAGGCAAGCGAAACTCGTGTACGTCAGTTCGTTGCGGACGCATCTCACGAACTGCGTACCCCACTGGCGGCAATCCGTGGTTACGCTGAAGTGGCGCGTCGTGGTCGTGCACAGGCTCCGCCGGATGTTGCTCACGCTCTGCGTCGCGTTGAATCTGAATCCGTTCGTATGACCAGCCTGGTTGATGATCTGCTGCTTCTGGCGCGTCTGGACTCTGGTCGTCCACTGGTTTCTGAACCGGTTGATCTGTCCGCACTGGTGGTTGATGCTGTTAGCGATGCTCACGTTGCAGGTCCGGAACAGCGTTGGCAGCTGGATCTGCCGGATGAAGCGGTTGAAGTTTCTGGCGATGCAGCTCGTTTGCACCAGGTTGTTACCAACCTGCTGACCAACGCTCGTGTACACACTCCACCGGGCACCACCGTTACTACTACTCTGCGTGCTGAACCGGACACCGCAGTACTGACCGTAGCGGATGACGGTCCGGGTATTCCAGCTGAACTTCAGCCAGAAGTCTTCGAACGTTTCGCACGTGGTGACTCTTCTCGTTCTCGTGCTCACGGTTCTACTGGTCTGGGTCTGGCGATCGTTGCAGCAGTTGTTGATGCTCACCACGGTCGTGTTGAAGTTGACAGCCGTCCAGGTCGTACTCTGTTCACCGTT

CGTCTTCCGGTGCACACCGGTGCTACTACCTCTGCA

## **Text S4** Optimized sequence of MechNtrC

ATGGTTACTGATGGTCACGCATTGCCAGGTCGTGTTGAACTCCGTCGTCCAGACGGTGGTCCGGTTCGTGTTCTGGTTGTGGATGACGAACCGACTCTGACCGATCTGCTGGCTATGGCTCTGCGTTACGAAGGTTGGCAGGTGGCAACCGCAGGTAACGGTATGGCGGCGCTGTCTAGCGCTCGTCAGGTTCGTCCGGATGTTGTTGTACTGGACGTTATGTTGCCGGACCTGGACGGTTTCCAGGTTCTGCGTCGTTTGCGTGAACAGTCTCCGAACGTTCCGGTACTGTTTCTGACCGCTCGTGATGCCGTTCAAGATCGTATCGCGGGTCTGACCGTAGGTGGTGACGACTACGTTACCAAACCGTTCAGCCTGGAAGAAGTTATCGCTCGTCTGCGTGCACTGCTGCGTCGTGCGGGTCTGGCAGTGGCTGCACGTGAAGACGCAGTTCTGACCGTTGGTGACCTGTCTCTGGATGAAGACAGCCACGAAGTTCGTCGTGGTGACGACCTGATCACTCTGACCGCGACCGAATTTGAACTGCTGCGTTACCTGATGCGTAATCCGCGTCGTGTTCTGAGCAAAGCGCAGATTCTGGACCACGTTTGGAACTACGATTTCGGTGGTCAGGCGAACGTTGTAGAACTGTACATCTCTTACCTGCGTAAGAAGATCGATGCTGGTCGTGAACCGATGATCCACACCTTGC

GTGGTGCAGGTTACGTTCTGAAACCGGCGCCACTCGAG

## **Table S1** The strains used in the experiment, their characteristics and origin

| **Strains** | **Characteristics** | **Source** |
| --- | --- | --- |
| *E.coli* BL21(DE3) | F- *ompT hsdSB* (*rB- mB-*) *gal dcm* (DE3) | Laboratory purchase |
| *E.coli* BL21(DE3)/pET-29a(+) | Control group for heterogeneous protein expression, Kan^r^ | Laboratory construction |
| *E.coli* BL21(DE3)/pET-29a(+)-*MechNtrB* | MechNtrB-expressing strain harboring the plasmid of pET-29a(+)-*MechNtrB*, kan^r^ | Laboratory construction |
| *E.coli* BL21(DE3)/pET-29a(+)-*MechNtrC* | MechNtrC-expressing strain harboring the plasmid of pET-29a(+)-*MechNtrC*, kan^r^ | Laboratory construction |
| Yeast AH109 | *MATa, trp1-901, leu2-3, 112, ura3-52, his3-200, gal4Δ, gal80Δ, LYS2::GAL1_UAS_-GAL1_TATA_-HIS3, MEL1 GAL2_UAS_-GAL2_TATA_-ADE2, URA3::MEL1_UAS_-MEL1_TATA_-lacZ* | Laboratory purchase |
| AH109/pGBT9 | Yeast two-hybrid strain, AH109 harboring the plasmid of pGBT9 | Laboratory construction |
| AH109/pGBT9-*MechNtrB* | Yeast two-hybrid strain, AH109 harboring the plasmid of pGBT9-*MechNtrB* | Laboratory construction |
| AH109/pGBT9-*AK2* | Yeast two-hybrid strain, AH109 harboring the plasmid of pGBT9-*AK2* | Laboratory construction |
| AH109/pGBT9+pGAD10 | Negative control for yeast two-hybrid, AH109 harboring the plasmid of pGBT9 and pGAD10 | Laboratory construction |
| AH109/pGBT9-*MechNtrB*+pGAD10-*MechNtrC* | Yeast two-hybrid experimental group, AH109 harboring the plasmid of pGBT9-*MechNtrB* and pGAD10-*MechNtrC* | Laboratory construction |
| AH109/pGBT9-*AK2*+pGAD10-*AIF* | Positive control for yeast two-hybrid, AH109 harboring the plasmid of pGBT9-*AK2* and pGAD10-*AIF* | Laboratory construction |
| *E.coil* ET12567/(pUZ8002) | *tra* gene encoding the transfer protein, Kan^r^, Chlo^r^ | Presented by teacher Yunchang Xie |
| *E.coil* DH5α | F-*φ80lacZm15 recA1 endA1 gyrA96* thi-1 *hsdR17* (rK-,mK-) *supE44 relA1 deoR* △*(lacZYA-argF) U169* | Laboratory purchase |
| *Micromonospora.echinospora* DSM 43816 | Can produce gentamicin complex, Kan^r^ | Laboratory purchase |
| *M.echinospora*/pSET152AKEx-*MechNtrB* | MechNtrB-overexpression strain | Laboratory construction |
| *M.echinospora*/pSET152AKEx-*MechNtrC* | MechNtrC-overexpression strain | Laboratory construction |

## **Table S2** The plasmids used in the experiment, their characteristics and origin

| **plasmids** | **Characteristics** | **Source** |
| --- | --- | --- |
| pET-29a(+) | Protein heterogenous expression vector, Kan^r^ | Laboratory purchase |
| pET-29a-*MechNtrB* | MechNtrB expression vector, pET-29a(+) with *MechNtrB*, Kan^r^ | Laboratory construction |
| pET-29a-*MechNtrC* | MechNtrC expression vector, pET-29a(+) with *MechNtrC,* Kan^r^ | Laboratory construction |
| pGBT9 | Bait vector for yeast two-hybrid, Amp^r^, | Laboratory purchase |
| pGBT9-*AK2* | Yeast two-hybrid, Bait vector pGBT9 with *AK2*, Amp^r^ | Laboratory purchase |
| pGBT9-*MechNtrB* | Yeast two-hybrid, Bait vector pGBT9 with *MechNtrB*, Amp^r^ | Laboratory construction |
| pGAD10 | AD vector for yeast two-hybrid, Amp^r^ | Laboratory purchase |
| pGAD10-*AIF* | Yeast two-hybrid, AD vector pGAD10 with *AIF*, Amp^r^ | Laboratory purchase |
| pGAD10-*MechNtrC* | Yeast two-hybrid, AD vector pGAD10 with *MechNtrC,* Amp^r^ | Laboratory construction |
| pSET152AKEx | Plasmid pSET152 added with erythromycin strong promoter | Presented by teacher Yunchang Xie |
| pSET152AKEx-*MechNtrB* | Conjugation transfer plasmid pSET152AKEx with *MechNtrB* | Laboratory construction |
| pSET152AKEx-*MechNtrC* | Conjugation transfer plasmid pSET152AKEx with *MechNtrC* | Laboratory construction |

## **Table S3** Primers for experiments

| **Primers name** | **Primer sequence (5^'^→3^'^）** |
| --- | --- |
| MechNtrB (Yeast two-hybrid)-F | AAAAGTCGACATGACCGGTTGGAGCCT |
| MechNtrB (Yeast two-hybrid)-R | AAAAGCGGCCGCTGCAGAGGTAGTAGCACCGG |
| MechNtrC (Yeast two-hybrid)-F | AAAACTCGAGATGGTTACTGATGGTCACGC |
| MechNtrC (Yeast two-hybrid)-R | AAAAGAATTCTGGCGCCGGTTTCAGAA |
| MechNtrB (overexpression)-F | GGAATTCCATATGACCACGGTGGCGCT |
| MechNtrB (overexpression)-R | CTAGTCTAGAGTGAACCGGCAGACGTAC |
| MechNtrC (overexpression)-F | GGAATTCCATATGGTGACCGACGGGCAT |
| MechNtrC (overexpression)-R | CTAGTCTAGACGGGGCCGGCTTGA |
| 16S-F | TGCTTAACACATGCAAGTCGAG |
| 16S-R | GTTATCCCAAAGCCTAGGGCAG |
| Glutamine synthetase -F | GCGATACCTCAAGAACGAGGAC |
| Glutamine synthetase -R | GAGGAAGACCAGACCGGTGG |
| Glutamate synthetase -F | ATCATGATCCAGGTGCCGGAC |
| Glutamate synthetase -R | GAGGAAGACCAGACCGGTGG |
| Glutamate dehydrogenase -F | TTCACCGCCGAGGAGATGCT |
| Glutamate dehydrogenase -R | GTGACGATCTCGACGACCGTGT |

## **Figure S1** Analysis of basic physicochemical properties of MechNtrB and MechNtrC proteins


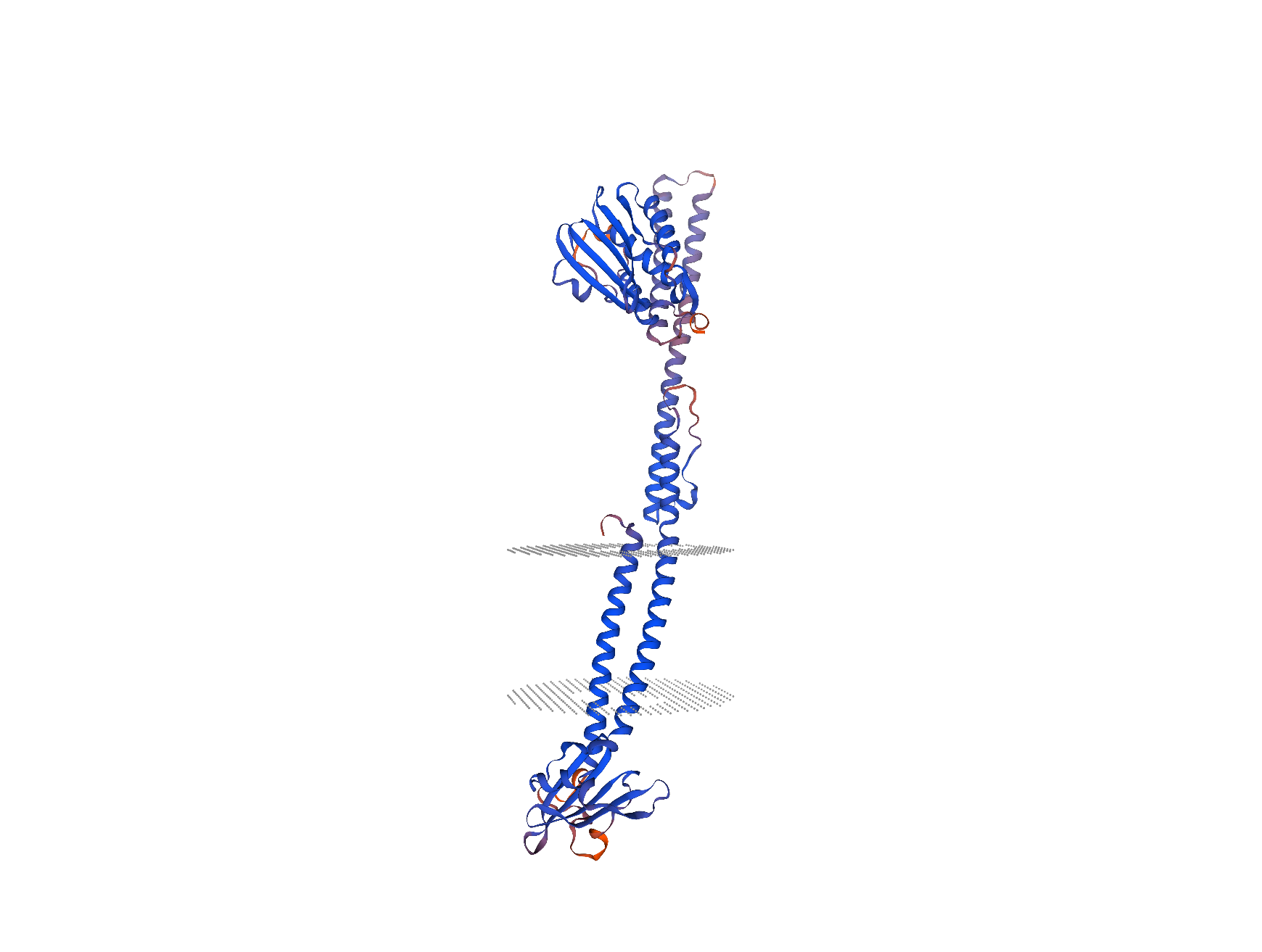

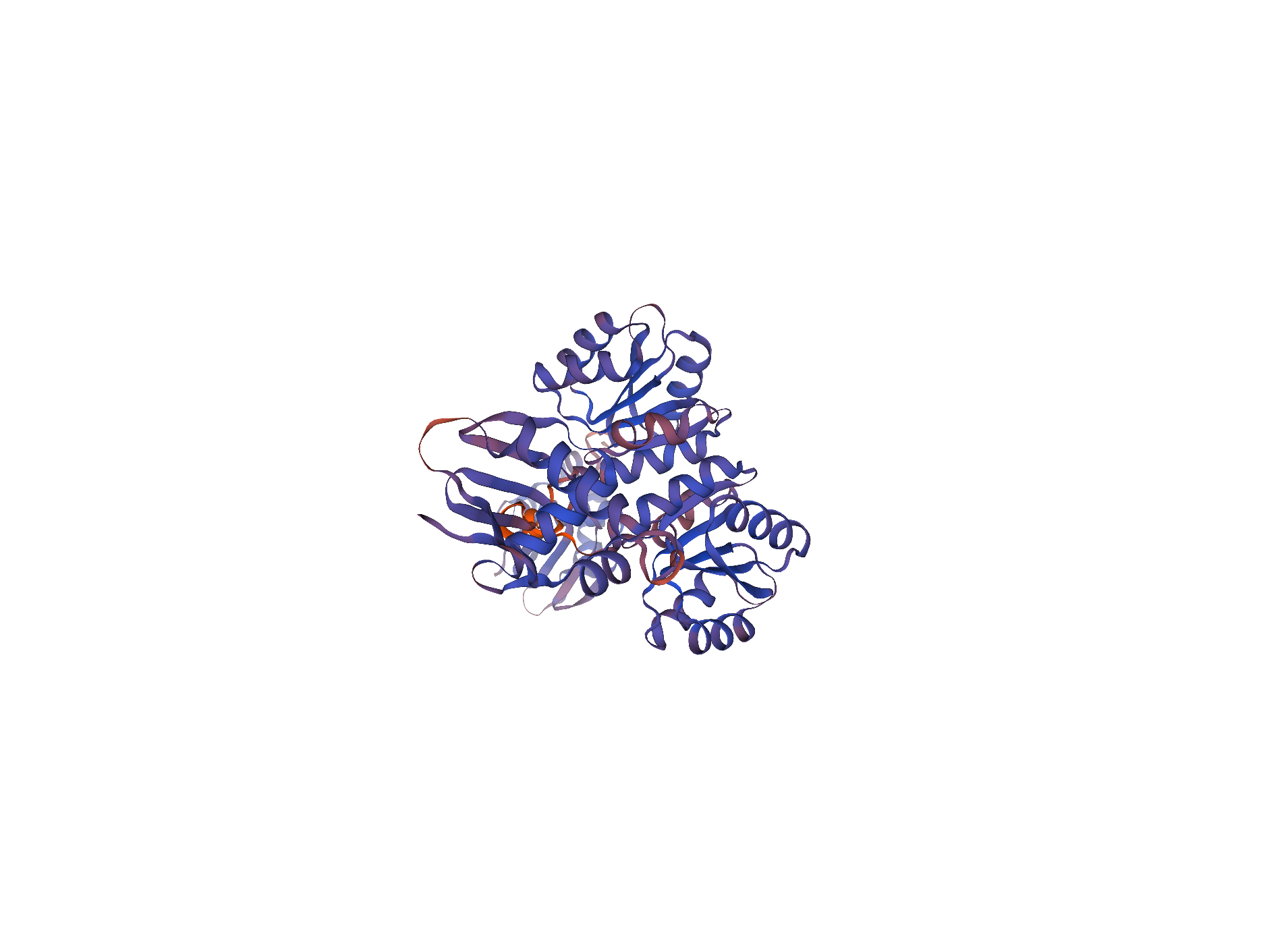


## **Figure S2** The tertiary structure model of the MechNtrB protein is above. The MechNtrC protein is below.

## **Figure S3** Ramachandran plots evaluation of MechNtrB and MechNtrC protein tertiary structure models


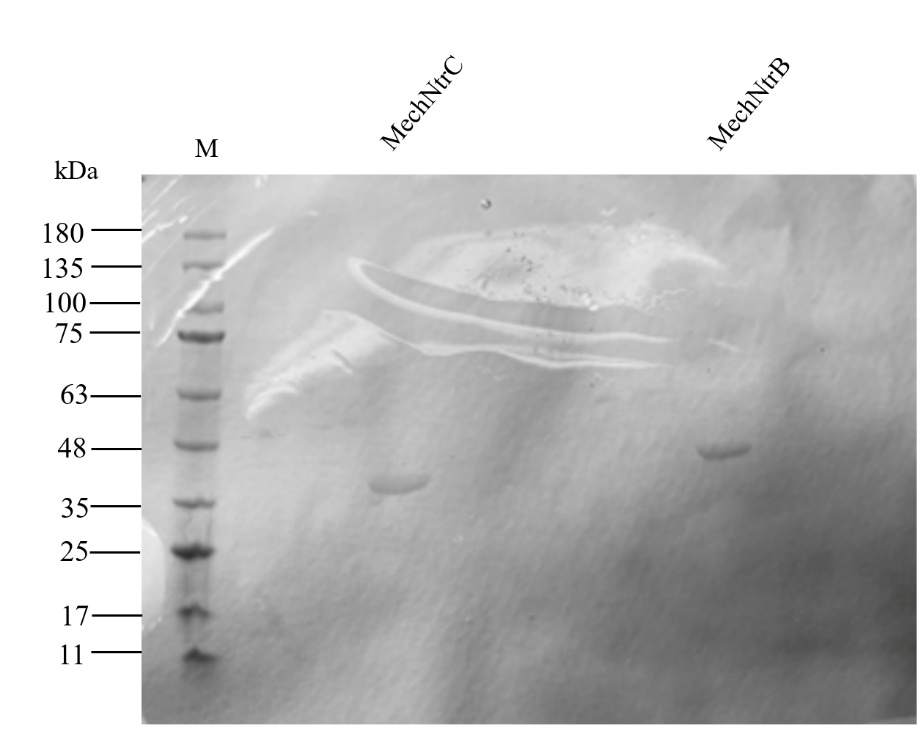


## **Figure S4** Coomassie Brilliant Blue (CBB)-stained gel of the dialyzed proteins.
